# Transformation and Evaluation of the MIMIC Database in the OMOP Common Data Model: Development and Usability Study

**DOI:** 10.2196/30970

**Published:** 2021-12-14

**Authors:** Nicolas Paris, Antoine Lamer, Adrien Parrot

**Affiliations:** 1 InterHop Paris France; 2 Univ. Lille, CHU Lille, ULR 2694 - METRICS: Évaluation des Technologies de santé et des Pratiques médicales Lille France

**Keywords:** data reuse, open data, OMOP, common data model, critical care, machine learning, big data, health informatics, health data, health database, electronic health records, open access database, digital health, intensive care, health care

## Abstract

**Background:**

In the era of big data, the intensive care unit (ICU) is likely to benefit from real-time computer analysis and modeling based on close patient monitoring and electronic health record data. The Medical Information Mart for Intensive Care (MIMIC) is the first open access database in the ICU domain. Many studies have shown that common data models (CDMs) improve database searching by allowing code, tools, and experience to be shared. The Observational Medical Outcomes Partnership (OMOP) CDM is spreading all over the world.

**Objective:**

The objective was to transform MIMIC into an OMOP database and to evaluate the benefits of this transformation for analysts.

**Methods:**

We transformed MIMIC (version 1.4.21) into OMOP format (version 5.3.3.1) through semantic and structural mapping. The structural mapping aimed at moving the MIMIC data into the right place in OMOP, with some data transformations. The mapping was divided into 3 phases: conception, implementation, and evaluation. The conceptual mapping aimed at aligning the MIMIC local terminologies to OMOP's standard ones. It consisted of 3 phases: integration, alignment, and evaluation. A documented, tested, versioned, exemplified, and open repository was set up to support the transformation and improvement of the MIMIC community's source code. The resulting data set was evaluated over a 48-hour datathon.

**Results:**

With an investment of 2 people for 500 hours, 64% of the data items of the 26 MIMIC tables were standardized into the OMOP CDM and 78% of the source concepts mapped to reference terminologies. The model proved its ability to support community contributions and was well received during the datathon, with 160 participants and 15,000 requests executed with a maximum duration of 1 minute.

**Conclusions:**

The resulting MIMIC-OMOP data set is the first MIMIC-OMOP data set available free of charge with real disidentified data ready for replicable intensive care research. This approach can be generalized to any medical field.

## Introduction

Intensive care units (ICUs) are designed to provide comprehensive support to the most severely ill patients in a hospital [1]. Mortality is typically high among these patients, both during and after the hospital stay [2]. Understanding the effects of interventions on patient outcomes remains a challenge due to the heterogeneity of patients, complexity of disease, and variation in care patterns. Intensivists use a limited level of evidence to guide decision making [3], whereas ICUs are a high-density environment for data production.

With the increasing adoption of electronic health record (EHR) systems around the world leading to large amounts of clinical data [4] and the development of data mining, innovation through data reuse is likely to play an important role in clinical medicine [5]. Indeed, based on important medical information, expectations are to improve clinical outcomes and practices, enable personalized medicine and guide early warning systems, and also easily enroll a large, multicenter cohort, while minimizing costs [6,7].

The Medical Information Mart for Intensive Care (MIMIC)-III is a high-granularity data set of over 60,000 intensive care stays and 46,000 unique patients from 2 successive ICU systems at the Beth Israel Deaconess Medical Center in Boston, admitted from 2001 to 2012 [8]. It is the first ICU database available for free, and it has been intensively used in research, resulting in more than 300 international publications. However, its monocentric nature makes it difficult to generalize findings to other ICUs.

For Kahn et al [9], “Database modelling is the process of determining how data are to be stored in a database.” It specifies data types, constraints, relationships, and metadata definitions and provides a standardized way to represent resources/data and their relationships. Some studies have shown that using a common data model (CDM) by standardizing the structure (data model) and concepts (terminological model) of the database allows larger-scale multicenter research and exploitation of rare diseases or rare events and catalyzes research by sharing practices, source code, and tools [10,11]. However, some studies have shown that the results are not fully reproducible from one CDM to another [12] or from one center to another [13]. Some approaches argue that keeping the local conceptual model [14] and the local structural model [15] leads to better results. On the one hand, keeping MIMIC in its specific form will not solve the limitation for multicenter research, but on the other hand, a fully standardized form would introduce other disadvantages, such as loss of data and lower computational performances. The ideal solution is probably in between to allow local or standardized analysis, depending on the research question.

The Observational Medical Outcomes Partnership (OMOP) CDM is a data model originally designed for multicenter research related to adverse drug events, which has been now extended to medical, laboratory, and genomic cases. OMOP provides structural and conceptual models relying on reference terminologies, such as Systematized Nomenclature of Medicine (SNOMED) for diagnostics, RxNORM for drugs, and Logical Observation Identifiers Names and Codes (LOINC) for laboratory results. Several examples of databases transformed into OMOP have been published [16-18], and OMOP stores more than half a billion patient records from around the world [19,20]. The OMOP conceptual model is based on a closure table pattern [21] capable of ingesting any simple, hierarchical, and also graph terminologies, such as SNOMED. In addition to local terminologies, OMOP defines and maintains a set of standard terminologies to be mapped unidirectionally (local to standard) by implementers. Although OMOP has proven its reliability [22], the concept mapping process is known to have an impact on results [23] and the application of the same protocol on different data sources leads to different results [13]. This shows the importance of keeping local terminologies so that local analysis is still possible. Previous preliminary work has been done on the translation of MIMIC into OMOP [24]. This work remains to be refined and updated for proper evaluation.

When comparing different CDMs [10,25], OMOP obtained the best results for completeness; integrity; flexibility; simplicity of integration; implementability for a wider coverage of the structural and conceptual model; a more systematic analysis, thanks to an analytical library and to visualization tools; and easier access to data through SQL queries. In terms of a conceptual approach, OMOP offers a broader set of standard concepts. In terms of a structural CDM, it is rigorous in how data should be loaded into specific tables, while other CDMs, such i2b2, are flexible with a general table that solves all data domains. This rigorous approach is necessary for standardization. Previous work has been performed to load MIMIC-III into i2b2 [26]; however, the work could not be finalized due to the tricky concept mapping to standard terminologies tasks. OMOP has the advantage of not making the terminology-mapping step mandatory by keeping the local codes accessible to analysts. Compared to the Fast Healthcare Interoperability Resources (FHIR) [27], OMOP performs better as a conceptual CDM because the FHIR resources currently do not specify the terminology to be used for most of the attributes. The OMOP relational model can be materialized in csv format and stored in any relational database, while the FHIR uses json files and needs some processing and higher skills to exploit. Among the above models, OMOP is the best candidate to overcome the MIMIC limitations mentioned earlier.

Our paper was guided by the 2 following objectives: (1) transforming MIMIC into OMOP in terms of the time needed, skills required, and quality of the result and (2) evaluating the resulting data set to support efficient, shareable, and real-time analysis.

## Methods

### Data

The majority of source code was implemented in PostgreSQL 9.6.9 (Postgres) because it is the primary support for the MIMIC database. It also allows the community to reproduce our work on limited resources without licensing costs and benefit from recent Postgres improvements in the data processing area. Some elaborated data transformations have been implemented as Postgres functions.

OMOP CDM version 5.3.3.1 (OMOP) tables were created from the provided scripts, with some changes documented in our scripts. OMOP defines 15 standardized CLINICAL data tables, 3 HEALTH system data tables, 2 HEALTH ECONOMICS data tables, 5 tables for DERIVED elements, and 12 tables for standardized VOCABULARY. The VOCABULARY tables were loaded from concepts downloaded from Athena [28], and the clinical and derived tables were loaded from MIMIC.

MIMIC-III version 1.4.21 (MIMIC) was also loaded into Postgres with the provided scripts. A subset of 100 patients over the 46,000 total MIMIC patients was selected based on their broad representativeness in the database and was cloned into a second instance to serve as a light and representative development set.

### Structural Mapping

The *structural mapping* aimed at moving the MIMIC data to the right place in OMOP, with some data transformations. It was organized into 3 phases: conception, implementation, and evaluation.

The *conception* phase consisted of looping over each MIMIC table and choosing an equivalent location in OMOP for each column. In general, both projects were appropriately documented, but in several cases, we needed some clarification from MIMIC contributors on the dedicated MIMIC git repository [29] or from the OMOP community forum [30]. Some trickier choices have been discussed in the MIMIC-OMOP git repository [31] and can be tracked in the commit logs.

The *implementation* was done through an extract-transform-load (ETL) process that consisted of Postgres scripts to extract information from the source or concept mapping tables and then transform it and load it into an OMOP target table. The scripts were managed sequentially through a main program. As a last resort, some modifications to the structural model of OMOP were made. A dedicated script recaps all of them and contains columns name modifications, new columns, column type modifications, or database indexing modifications. In particular, each source table has been given a unique global sequence incremented from 0, which serves as the primary key and links to the OMOP target tables. As a result, every record was uniquely identified, allowing us to chain the information with OMOP, while simplifying the maintenance of primary/foreign keys.

Although *evaluating* a structural model is difficult [32], several papers have attempted to assess the quality of the CDM [9,25]. The criteria developed by Khan et al [9], which refer to the Moody and Shanks metrics [32], were adapted to assess the quality of the data transformation (Table 1).

**Table 1:** Transformation quality evaluation metrics.

Data model dimension	Description
Completeness: structural mapping	Domain coverage: coverage of sources domains that are accommodated by the standard OMOP^a^ model
Completeness: conceptual mapping	Data coverage: coverage of sources data concepts that mapped to the standard OMOP concept
Integrity	“Meaningful data relationships and constraints that uphold the intent of the data's original purpose” [9]
Flexibility	The ease to expand the standard model for new datatypes and concepts
Integration	The capacity of the standard model to use multiple terminologies and link them to standard ones
Implementability	The stability of the models, the community, and the cost of adoption
Understandability	The ease of the standard model to be understood
Simplicity	The ease of querying the standard model (the model should contain the minimum of concepts and relationship)

^a^OMOP: Observational Medical Outcomes Partnership.

In addition to the Moody and Shanks metrics, we provided a set of controls to guarantee correct transformation. To compare overall statistics, some SQL queries were set up to compare MIMIC and MIMIC-OMOP, and we provided basic characterizations of the populations. All tables were covered and tested through simple counts, aggregate counts, or distribution checks. We estimated the loss of information during the ETL process by measuring the percentage of both columns and rows lost in the process, as other previous studies have done [17]. It is important to note that we chose not to keep irrelevant information: for example, some rows are known to be invalid in MIMIC or some information is redundant. Each ETL script was tested using pgTAP, a unit testing framework for Postgres. Each unit test script checked whether a particular OMOP target table was correctly loaded. Integrity constraints (primary keys, foreign keys, nonnull columns) were included to apply integrity checks at ETL run time. The last part of the structural evaluation was Achilles software. It is open source analysis software produced by Observational Health Data Sciences and Informatics (OHDSI) [33]. Like many previous authors, we used Achilles to assess data quality [34]. This tool is used for data characterization, data quality assessment (Achilles Heel), and health observation data visualization. All the resulting tables are presented in the Results section.

### Conceptual Mapping

The *conceptual mapping* aimed at aligning the MIMIC local terminologies to OMOP's standard ones. It consisted of 3 phases: integration, alignment, and evaluation.

The *integration* phase consisted of loading both types of terminologies into the OMOP vocabulary tables. The OMOP terminologies are provided by the Athena tool and were loaded with the associated programs. We used export with all terminologies without licensing limitations. The local terminologies were extracted from the multiple MIMIC tables and loaded into the OMOP CONCEPT table. When possible, relevant information from the original MIMIC tables was concatenated in the *concept_name* column. MIMIC local concepts were loaded with a *concept_id* identifier starting from 2 billion. In the OMOP CONCEPT table, MIMIC concepts could be distinguished with the *vocabulary_id* identifier equal to “MIMIC code” and a *domain_id* identifier targeting the OMOP table in which the corresponding data were stored. This domain information was used in the ETL to send the information to the proper table. Following OMOP documentation, the conceptual mapping has to be performed before the structural mapping because the nature of the standard OMOP concepts guides in which table (domain) the information should be stored.

The *alignment* phase, aimed at standardizing local MIMIC codes into standard OMOP codes, had 4 distinct cases. In the first case, some MIMIC data were, by chance, already coded according to standard OMOP terminologies (eg, LOINC laboratory results) and, therefore, the standard and local concepts were the same. In the second case, MIMIC data were not coded according to the standard OMOP terminologies but the mapping was already provided by OMOP (eg, *International Classification of Diseases, 9th Revision* [ICD9]/Systematized Nomenclature of Medicine-Clinical Terms [SNOMED-CT]), so the domain tables were loaded accordingly. In the third case, terminology mapping was not provided, but it was small enough to be done manually in a few hours (eg, demographic status, signs, and symptoms). In the fourth case, terminology mapping was not provided and consisted of a large set of local terms (admission diagnosis, drugs). Next, only a subset of the most represented codes was manually mapped.

We chose to use simple SQL queries that were flexible enough to be queried on demand or to generate a prefilled csv with the best matches. We used Postgres full-text ranking features and linked local and standard candidates with a rating function based on their labels. This work was performed under the control of an intensivist.

The *evaluation* phase was both quantitative and qualitative. The quantitative evaluation measured the completeness of our work: the percentage of local concepts that were mapped to standard concepts. The qualitative evaluation assessed the correctness. For newly generated mappings, this consisted of manually tagging each mapping with a score between 0 and 1 and eventually writing a commentary on each mapped concept. In case where the mapping was provided by automatic OMOP terminology mapping, the evaluation was performed on a subset of concepts manually picked within each terminology.

### Data Analytics

Beyond the model transformation and with regard to the OMOP standardization process, we performed some analysis. MIMIC provides a large number of SQL scripts for preprocessing and normalizing data, calculating derived scores, and defining cohorts. Some of them were implemented on top of the OMOP format to load the OMOP-derived tables.

A set of *general denormalized* tables was built on top of the original OMOP format and had the *concept_name* related to the *concept_id* columns. The CONCEPT table is a central element of OMOP, and therefore, it was involved in many joins to obtain the concept label. By precalculating the joins with the CONCEPT tables, the denormalized tables rendered faster calculation and simplified SQL queries.

In addition, a set of *specialized analytical tables* was built, in addition to the original OMOP tables. The MICROBIOLOGICALEVENTS table was a reorganization of the MEASUREMENT table data of microorganisms and associated susceptibility testing antibiotics. It was based on the MIMIC MICROBIOLOGICALEVENTS table. The ICUSTAYS table allowed us to quickly determine the patients admitted in resuscitation and was inspired by the MIMIC ICUSTAYS tables.

The OMOP NOTE_NLP table was originally designed to store the final or intermediate derived information and metadata from clinical notes. When definitive, the extracted information is intended to be moved to the dedicated domain or table and then reused as regular structured data. When the information is still intermediate, it is stored in the NOTE_NLP table and can be used for later analysis. To populate this table, we provided 2 information extraction pipelines. The first pipeline extracted numerical values, such as weight, height, body mass index, and left ventricular cardiac ejection fraction, from medical notes with a SQL script. The resulting structured numerical values were loaded into the measurement or observation tables according to their domain. The second pipeline *section extractor*, based on the Apache Unstructured Information Management Architecture (UIMA) framework, divided notes into sections to help analysts choose or avoid certain sections of their analysis. Section templates (eg, “Illness History”) were automatically extracted from text with regular expressions and then filtered to keep only the most frequent (frequency >1%).

A 48-hour open access datathon was set up in the Assistance Publique des Hopitaux de Paris (Paris AP-HP) in collaboration with the Massachusetts Institute of Technology (MIT), once the MIMIC-OMOP transformation was ready for research. This datathon was organized to evaluate OMOP as an alternative data model for accessing and analyzing MIMIC data during a real event. Scientific questions were prepared in an online forum where participants could introduce themselves and propose a topic or choose an existing one. OMOP was loaded into Apache HIVE 1.2.1 in ORC format. Users had access to the ORC data set from a web interface Jupyter Notebooks with Python, R, or Scala. A SQL web client allowed teams to write SQL queries from Presto to the same data set. The hadoop cluster was based on 5 computers with 16 cores and 220 GB of RAM. The MIMIC-OMOP data set was loaded from a Postgres instance to HIVE through Apache SQOOP 1.4.6 directly in ORC format. Participants also had access to the Schemaspy database physical model to access the OMOP physical data model with both table/column comments and key primary/foreign relationships materializing the relationships between the tables. All queries were logged.

## Results

### Data Transformation

All transformation processes are freely accessible to the public via the MIMIC-OMOP git repository maintained by MIT-LCP [8]. The git repository centralizes the various resources of this work, such as documentation, source code, unit tests, and questioning examples, discussions, and problem issues. It also indicates web resources, such as the physical data model for MIMIC and OMOP data sets and the Achilles Heel web client.

The MIMIC-OMOP conversion was performed by 2 developers (a data engineer and an intensivist) for 500 hours. This included ETL, git documentation, concept mapping, contributions, and unit tests. ETL (with unit tests and generation of ready-to-load archive) on a subset of 100 patients lasted 5 minutes and enabled fast development cycles. ETL lasted 3 hours to process the whole MIMIC database. The resulting csv archive was almost the same size as the original archive, and MIMIC-OMOP was also the same size as MIMIC once loaded and indexed into Postgres.

### Structural Mapping

The results of the structural mapping are presented in Table 2. Of the 37 OMOP tables, the ones related to hospital costs were not applicable, some tables related to derived data were not populated, and some tables related to vocabulary were preloaded with terminology information. The 26 tables of MIMIC were dispatched into 19 OMOP tables. The reduced number of tables resulted from the differences in the design of both models. OMOP stores all the terminologies in 1 table, whereas MIMIC has 1 table for each terminology. In addition, the same applies for facts data, which are grouped by nature in OMOP, while MIMIC tables are more specialized and respect the source EHR's design. For example, the MEASUREMENT table gathers measured information and combines 4 source tables, resulting in 365,181,104 rows, which is 20% more than the largest MIMIC table. To some extent, this is a regression in terms of performance.

**Table 2:** MIMIC^a^-OMOP^b^ data flows.

OMOP tables	Number of rows (n)	MIMIC tables
CARE_SITE	93	transfers, service
COHORT_ATTRIBUTE	228,379	callout
CONCEPT	30,344	d_cpt, d_icd_procedures, d_items, d_labitems
CONDITION_OCCURRENCE	716,595	admissions, diagnosis_icd
DEATH	14,849	patients, admissions
DRUG_EXPOSURE	24,934,751	prescriptions, inputevents_cv, inputevents_mv
MEASUREMENT	365,181,104	chart/lab/microbiology/in/output events
NOTE	2,082,294	noteevents
NOTE_NLP	16,350,855	noteevents
OBSERVATION	6,721,040	admissions, chartevents, datetimevvents, drgcodes
OBSERVATION_PERIOD	58,976	patients, admissions
PERSON	46,520	patients, admissions
PROCEDURE_OCCURRENCE	1,063,525	cptevents, procedureevents_mv, procedure_icd
PROVIDER	7567	caregivers
SPECIMEN	39,874,171	chartevents, labevents, microbiologyevents
VISIT_OCCURRENCE	58,976	admissions
VISIT_DETAIL	271,808	admissions, transfers, service

^a^MIMIC: Medical Information Mart for Intensive Care.

^b^OMOP: Observational Medical Outcomes Partnership.

Two important tables are provided by OMOP to model the relationship between the data: CONCEPT_RELATIONSHIP and FACT_RELATIONSHIP. We used them to bind the drugs into a solution for microbiology/antibiograms and for VISIT_DETAIL/CARE_SITE links. The following SQL query (Textbox 1) shows how a microorganism is linked to its susceptibility test by a FACT_RELATIONSHIP and illustrates the flexibility of the model. However, this flexibility affects the simplicity and the performance of the model by increasing the number of joins within SQL queries.

**Original table microbiology SQL query.**

SELECT measurement_source_value

, value_as_concept_id

, concept_name

FROM measurement

JOIN concept resistance

ON value_as_concept_id = concept_id

JOIN fact_relationship

ON measurement_id = fact_id_2

JOIN

(

SELECT measurement_id AS id_is_staph

FROM measurement

WHERE

measurement_type_concept_id = 2000000007

-- 'Labs - Culture Organisms'

AND value_as_concept_id = 4149419

-- 'Staph aureus coag +'

AND measurement_concept_id = 46235217

-- 'Bacteria identified in Blood product

unit.autologous by Culture'

) staph ON id_is_staph = fact_id_1

WHERE TRUE

AND measurement_type_concept_id = 2000000008

-- 'Labs - Culture Sensitivity'

Table 3 presents the basic characterization of the MIMIC-OMOP population and assesses the overall quality of structural mapping. Fortunately, most statistics remain similar between the 2 versions, with few differences. Table 3 shows that MIMIC contains 61,532 intensive care stays, while OMOP contains 71,576 intensive care stays. This represents a 16% increase in stays.

**Table 3:** Baseline characteristics of MIMIC^a^ versus OMOP^b^.

Items		MIMIC	MIMIC-OMOP
**Overall**			
	Persons (n)	46,520	46,520
	Admissions (n)	58,976	58,976
	ICU^c^ stays (n)	71,575	61,532
	Female gender, n (%)	20,399 (43.85)	20,399 (43.85)
**Age (N=58,976)**			
	Mean	64 years, 4 months	64 years, 4 months
	0-5 years, n (%)	8110 (13.75)	8110 (13.75)
	6-15 years, n (%)	1 (0.001)	1 (0.001)
	16-25 years, n (%)	1434 (2.43)	1434 (2.43)
	26-45 years, n (%)	5962 (10.11)	5962 (10.11)
	46-65 years, n (%)	17,375 (29.46)	17,375 (29.46)
	66-80 years, n (%)	15,793 (26.78)	15,793 (26.78)
	>80 years, n (%)	10,301 (17.47)	10,301 (17.47)
**Other characteristics**			
	Emergency, n	42,071	42,071
	Elective, n	7706	7706
	Surgical patients, n	19,246	19,246
	Length of hospital stay, days, median (Q1-Q3)	6.46 (3.74-11.79)	6.59 (3.84-11.88)
	Length of ICU stay, days, median (Q1-Q3)	2.09 (1.10-4.48)	1.87 (0.95-3.87)
	Mortality in ICU, n (%)	5814 (9)	5815 (9)
	Mortality in hospital, n (%)	4511 (7)	4559 (6)
	Lab measurements per admissions, mean	478	678
	Procedures per admission, mean	4.6	4.6
	Drugs per admission, mean	82.8	82.8
	Exit diagnosis per admission, mean	11	11

^a^MIMIC: Medical Information Mart for Intensive Care.

^b^OMOP: Observational Medical Outcomes Partnership.

^c^ICU: intensive care unit.

By design, MIMIC aggregates information from various systems. Thus, the transfer information is divided into several tables, such as ADMISSIONS, TRANSFERS, and also ICUSTAYS, while OMOP centralizes this information in VISIT_DETAIL. We also added emergency stays as a normal location for patients throughout their hospital stay (unlike what had been done by MIMIC). The ICUSTAYS MIMIC table was not transformed, because it derives from the TRANSFER table and we decided to assign a new VISIT_DETAIL row for each ICU stay (based on the TRANSFER table), while MIMIC prefers to assign a new ICU stay if a new admission occurs more than 24 hours after the end of the previous stay. This table also showed an increase in the number of laboratory measurements per admission. This is because MIMIC-OMOP gathers laboratory data from both the MIMIC-dedicated LABORATORY table and the CHARTEVENTS table, which is usually not considered for this purpose. For laboratory tests, we put a specimen (ie, a blood sample) for many laboratory results (because 1 blood sample can be used for several tests), and we decided to create as many rows of samples as laboratory tests because the information was not present in MIMIC. The same was true when date information was not provided (start/end _datetime} for DRUG_EXPOSURE).

As mentioned in Table 4, 20%-80% of the source columns were not retained. Almost all were redundant or provided derived information. The main concern was the loss of some timestamps. For example, the MIMIC CHARTEVENTS table provides the *storetime* and *charttime* columns, but OMOP only provides 1 column to store timestamps. Thus, the MIMIC \*storetime* column was eliminated during ETL, which was considered less valuable.

**Table 4:** Data lost.

Relationship	Rows lost, %	Columns lost, %
admissions	—^a^	30
callout	—	80
caregivers	—	50
chartevents	0.04	40
cptevents	—	60
datetimeevents	0.0001	50
diagnoses_icd	—	20
drugcodes	—	60
inputevents_cv	—	41
inputevents_mv	10.0	46
labevents	—	34
microbiologyevents	—	30
noteevents	0.04	19
outputevents	—	39
patients	—	50
prescriptions	—	16
procedureevents_mv	3.0	70
procedures_icd	—	40
services	—	34
transfers	—	47

^a^Not available.

As mentioned in the Methods section, incorrect entries were not kept in the process. Five MIMIC tables (INPUTEVENTS_MV, CHARTEVENTS, PROCEDUREEVENTS_MV, NOTEEVENTS, and DATETIMEEVENTS) had deleted rows in the ETL process. All of them were tagged in MIMIC as erroneous or cancelled.

A set of minor modifications of the OMOP table structure was made in order to fit the data. All character columns with limited length were modified to unlimited length since this could cause unpredictable truncation of content, while having no negative impact on the Postgres storage size or performance. The VISIT_OCCURRENCE and VISIT_DETAIL tables were corrected according to some discussions of the OHDSI forum. The NLP_NOTE table was extended with fields mentioned in online documentation but forgotten in the scripts. In addition, the *offset* column was divided into 2 integer-type columns because the offset term was a SQL reserved word and it made sense to fill the resulting *offset_begin* and *offset_end* columns with integer values.

All the PgTAP unit tests passed. Moreover, OMOP had a 100% match of the integrity constraints and the foreign key relationships of the data models. After 18 hours of computations, Achilles Heel issued 15 errors, 18 warnings, and 8 notifications. This result is good compared to other studies [27].

### Conceptual Mapping

The results of the conceptual mapping's completeness are presented in Table 5. We have often mapped many source concepts to a unique standard *concept_id* because MIMIC provides a large number of equivalent concepts. For example, MIMIC provides 6 distinct concepts for body temperature: temperature C, temperature C (calc), temperature F, temperature F (calc), temperature Fahrenheit, and temperature Celsius. All of them were mapped to the LOINC “Body temperature”, and numerical values were normalized.

**Table 5:** Terminology mapping coverage.

OMOP^a^ tables (domain)	Records, n	Mapped records, n (%)	Concept source, n	Mapped concepts source, n (%)
CARE_SITE	144	144 (100)	58	58 (100)
CONDITION_OCCURRENCE	716,595	644,936 (90)	6984	6565 (94)
DRUG_EXPOSURE	24,934,751	9,475,205 (38)	7398	4143 (56)
MEASUREMENT	40,141,521	29,303,310 (73)	1035	787 (76)
OBSERVATION	6,721,040	4,570,307 (68)	1440	1152 (80)
PERSON	93,040	93,040 (100)	43	43 (100)
PROCEDURE_OCCURRENCE	1,063,525	1,052,890 (99)	2203	2181 (99)
SPECIMEN	39,874,171	27,911,920 (70)	92	71 (77)
NOTE	2,082,294	2,082,294 (100)	15	15 (100)
VISIT_OCCURRENCE	176,928	176,928 (100)	34	34 (100)
VISIT_DETAIL	396,932	396,932 (100)	28	28 (100)

^a^OMOP: Observational Medical Outcomes Partnership.

OMOP's terminology coverage has already been rated as excellent [24]. We used the OMOP terminology mappings (National Drug Code [NDC]-RxNorm, ICD9-SNOMED, Common Procedural Terminology Fourth Revision [CPT4]-SNOMED) to standardize a consequent set of MIMIC nonstandard terminologies.

The automatic OMOP terminology mapping was evaluated by an intensivist. The results are in favor of good integration of the model. We checked 100 elements for each mapping used (NDC, ICD9, and CPT4). ICD9 and CPT4 were correctly mapped to SNOMED (100%). However, only 85% of NDCs were linked to a correct RxNorm code. This was partly due to an incorrect NDC drug code (from MIMIC) and partly because only 78% of NDC codes are mapped to RxNorm. Moreover, even if this does not seem to have affected our ETL, we know that some of ICD-9-CM codes can have a one-to-several match with SNOMED (28%) [35].

In several cases, OMOP had no suitable concepts for the ICU-specific cases. In particular, the VISIT_DETAIL table does not yet introduce relevant information and duplicate information from the VISIT_OCCURRENCE table. Therefore, we extended the concepts to track bed transfers and room transfers through *admitting_concept_id*, *discharge_to_concept_id*, or *visit_type_concept_id* columns. These added concepts were introduced with *concept_id* between 2 billion and 2.001 billion to distinguish them from OMOP concepts (0-2 billion) and MIMIC locals (>2.001 billion).

Some local concepts could not be mapped to standard ones. These unmapped concepts were linked with the *concept_id* = 0 and appeared in different cases. In the first case, the local concept has no equivalent in the standard vocabularies. In the second case, it has not yet been mapped and may have a standard equivalent. In the third case, the value is missing and cannot be mapped. In our opinion, although not all of these cases can be used for standard queries, they should have a different concept identifier in order to be treated differently (not just *concept_id* = 0). Some of the *domain_id* do not match the table name, and this makes sense because the OBSERVATION domain can be the MEASUREMENT table and vice versa. Although various types of information are stored in the MEASUREMENT table, the dedicated OMOP concepts for the *measurement_type_concept_id* column were not sufficient to distinguish them. Therefore, we added some *measurement_type* concepts (eg, Labs - Chemistry, Labs - Culture Organisms).

### Analytics

Some MIMIC raw information was transformed and added to match the structural model. The laboratory textual values were split into operators, numeric values, and units, when needed, with a dedicated Postgres stored procedure. The free text conditions were normalized and mapped to standard OMOP codes to meet the conceptual model.

As indicated in the Methods section, we provided many *derived values*. Common derived information was introduced and loaded: corrected serum calcium, corrected serum potassium, the P/F ratio, corrected osmolarity, and the Simplified Acute Physiology Score (SAPS) II.

*Denormalized derived* tables improved SQL query performance and verbosity. In addition, the resulting tables were much more human-readable, with the concept label directly in the table and greatly reduced joins. Therefore, a little denormalization greatly improved the analysts’ experience of the data model and simplicity by adding some redundancy in the data, while not interrupting existing SQL queries. Moreover, these normalized views were backward-compatible and remained standardized, allowing the creation of multicentric algorithms. We provided 2 examples of materialized specialized views derived from MICROBIOLOGYEVENTS and ICUSTAYS MIMIC that simplified the experience for scientists (Textbox 2). These results reflect the lack of simplicity of the model in its original form, but this can be easily overcome with such analytics tables. These results were in favor of good flexibility of the model, allowing us to store derived data.

**Optimized and denormalized microbiology table SQL query.**

SELECT antibiotic_source_value,

antibiotic_interpretation_concept_id,

antibiotic_interpretation_concept_name

FROM microbiology

WHERE

organism_concept_id = 4149419

-- 'Staph aureus coag +'

AND specimen_concept_id = 46235217

-- 'Bacteria identified in Blood product

unit.autologous by Culture';

The note section *extraction pipeline* resulted in 1200 sections that were collected and then manually filtered to exclude false positives; 400 similar groups were highlighted. The extracted sections were not mapped to standard terminologies, such as the LOINC clinical document ontology (CDO). The reason for this is that the LOINC CDO decided not to keep these sections up to date, considering that they are not widely used [36].

The Paris AP-HP organized a datathon with MIMIC-OMOP, in which 160 participants from 25 teams had 48 hours to undertake a clinical project using the MIMIC-OMOP database. They launched around 15,000 queries, with a maximum duration of 1 minute. They got an opportunity to create mixed teams: clinicians brought the issues that required data mining, as well as their data expertise, while data scientists judged the technical feasibility and finally implemented the various analyses needed. Writing standard queries (ie, with standard concepts) requires knowing the organization of relational models (SQL) and also mastering the graphical nature of certain terminologies, such as SNOMED-CT, in order to capture all potential codes that might be related to the one analysts think of first. Overall, the teams quickly mastered the OMOP model and managed to produce results at the end of the datathon. These results were in favor of good understandibility and simplicity of the model.

## Discussion

### Principal Results

In this paper, we presented the transformation of the MIMIC database into the OMOP CDM and its evaluation. The first major contribution of this study is to provide a freely accessible data set in OMOP format that could be useful to researchers. The second major contribution is to share with the OMOP community some useful transformations dedicated to intensive care that can be reused on any OMOP data set. The last contribution is to evaluate the implementation of MIMIC into the OMOP CDM.

### Lessons Learned

We observed that the OMOP CDM can be implemented at low cost and downstream of an existing architecture, since the scripts are freely available on the project's GitHub, for 8 different database management systems. The rationale of the data model can be understood through the numerous resources made available by the OHDSI community: tutorials, forums, working groups, and documentation. The structural mapping is carried out without difficulty as question marks can be raised with the community. The main difficulty remains the step of semantic mapping, especially in countries or institutions using local terminologies and vocabularies. Since the CDM model proposes to store both international and local vocabulary codes for each table, it is possible to start conducting studies using only the local codes. The mapping to the international codes can be carried out in a second phase, project by project, for the codes presented by each study. This will make it easier to spread out the difficulty of global mapping over thousands of codes.

### Data Transformation

The choice of a simple SQL-based ETL over dedicated ETL software has several advantages. SQL, as a unique language, factors both people's knowledge and computer resources, allowing analysts to become implementers and revise code or contribute to transformations. SQL was also used for semantic mapping, and OHDSI provides Usagi [37]. The use of csv format for sharing information is simple and universal. Both SQL and CSV are standard and target a large community (physicians, engineers, and analysts) with translational profiles and is compatible with multiple technologies.

The calculation time of ETL on the Postgres instance on a modest personal computer is compatible with community work where the collaborator can clone the source code and configure a development instance to reproduce or improve the work.

Choosing a public GitHub repository for documentation and source code support allows analysts to learn more about the project and also learn how to contribute. The highly active OMOP forum is full of details and training. In contrast, the implementation guide suffers from not being as detailed and maintained. We believe that the OMOP community would greatly benefit from a systematic and concise synchronization between the forum, mailing lists, source code repository, and end-user documentation.

Any data transformation is likely to generate bugs that can later have an impact on medical research. The foundations of the relational database management system (RDBMS), such as transactions, standardization, and integrity constraints, are integrated safeguards that have been useful throughout the process. In addition, the implemented unit tests ensure that past bugs are not repeated. An ideal but complex validation method would be to replicate existing MIMIC studies and ensure that the results are consistent across data models. The OHDSI Achilles tool completes our quality assessment. It is a surprisingly slow tool to process. The rules and their descriptions are difficult to understand. A more specific tool should be provided and described.

Another missing aspect is a set of quality tables for assessing and measuring data quality. MIMIC has a column to keep track of corrupted information. It would be interesting to be able to keep the disordered data in OMOP and enable research in the data cleaning/quality field. Although the OMOP-CDM provides rules to name columns, there are some mistakes, and we have to modify it. One the one hand, it is a problem for a CDM to contain errors, but on the other hand, it is easy to relay issues that are now corrected.

### Data Analytics

It is important that OMOP maintain a level of standardization in order to simplify ETL and make it consistent. However, once done, it makes sense to give access to scientific data through more denormalized and specialized tables. There are many concerns about OMOP's performance and optimization. However, there will never be a perfect multipurpose case table, and it is the responsibility of data scientists to build their own, simplified, specialized tables for their research and to respond effectively and clearly to their needs.

The derived data integrate quite well into OMOP. We used the NOTE_NLP table to store information derived from notes, the MEASUREMENT table to store derived numerical information, and the COHORT_ATTRIBUTE table to store derived scores. However, it is not yet clear whether derived data should be stored by domain or whether they should be stored in dedicated derived tables. We found that there are no tables to track the source and description of these data.

The pipeline notes' section extractor we used was based on the Apache UIMA framework. Although some methods already exist to extract medical sections [38], the prior work of describing sections was too complex, and we opted for a naive approach.

Last but not least, as noted in the Introduction section, a good CDM for the ICU would allow for near real-time early warning systems and inference modeling on fresh data. OMOP is clearly designed to provide a static data set and does not have real-time ingestion and data versioning control mechanisms like EHRs usually do. Analysis of static data sets is essential for reproducible results. However, when the algorithms need to be moved to the bedside, it is necessary to have fresh data and a way of re-identifying the patient that OMOP does not yet provide. That said, a solution such as the HL7 FHIR is a great way to implement real-time inference from EHR data, and that is how the FHIR and OMOP are complementary. This has already been studied but needs further optimization [39].

The MIT regularly organizes datathons using their open-access databases [40-43]. From a human point of view, these events enable teamwork and collaboration between different specialties (ie, physicians, computer scientists, statisticians, data scientists), which can benefit from each other's expertise. This time, the datathon was also an opportunity for these profiles to collaborate, and it allowed novices to be introduced to the OMOP CDM and its analytical tools. The critical point in the conducting of such an event is related to the IT architecture, which must allow dozens of users to run large queries at the same time and to share scripts and results. We used a platform similar to the one used by Celi et al [41], with several analytical tools (Jupyter Notebook, Python, R, Scala).

The datathon showed that distributed platforms with basic hardware provide SQL tools for online analytical processing (OLAP) with excellent performance that overcomes RDBMS weaknesses. Therefore, OLAP takes advantage of SQL language analysis functions, such as grouping, windowing, assembling, and mathematical functions, that are often missing in NoSQL databases. Although some are open source, these distributed technologies are not easily accessible; however, cloud-based solutions are increasingly affordable for researchers.

The real-life test of the datathon revealed the strong need to make the physical data model accessible, including comments on columns and tables, and we discovered that an open source tool called schemaspy is helpful. In addition, we found that the GitHub repository is the best place to document and interact with the community.

The OMOP model is powerful because it allows a broad spectrum of analysis from specialized local models to evidence-based statistical analysis in an easy-to-learn and accessible format. The major complexity of this model is intrinsically linked to the terminologies’ complexity with the use of its closure table [21].

Compared to the original MIMIC data model, working with OMOP offers the ability to write standard code and analyses that could benefit other international users.

The effectiveness of the OMOP model has some weaknesses because it seems to focus on consistency rather than performance. However, we have shown that it is easy to overcome these weaknesses and improve OMOP with design or technology optimization and a dedicated structure that ultimately remains a standard and is shareable because it derives from the original model.

### Conclusions

The transformation of MIMIC into OMOP required efforts that remain reasonable. It is and always will be a work in progress because standard concept mapping is an almost infinite process with constant improvements. Fortunately, the published version of MIMIC-OMOP is search ready and already offers the same scope of data as the original MIMIC version and even more with the derived data. It is publicly available on the GitHub repository and have been designed to be easily revised, copied, or enriched according to the OMOP or MIMIC philosophy by any users who know SQL.

## Abbreviations

CDM: common data model
CDO: clinical document ontology
CPT4: Common Procedural Terminology Fourth Revision
EHR: electronic health record
ETL: extract-transform-load
FHIR: Fast Healthcare Interoperability Resources
ICD9: International Classification of Diseases, 9th Revision
ICU: intensive care unit
LOINC: Logical Observation Identifiers Names and Codes
MIMIC: Medical Information Mart for Intensive Care
MIT: Massachusetts Institute of Technology
NDC: National Drug Code
OHDSI: Observational Health Data Sciences and Informatics
OLAP: online analytical processing
OMOP: Observational Medical Outcomes Partnership
RDBMS: relational database management system
SAPS: Simplified Acute Physiology Score
SNOMED: Systematized Nomenclature of Medicine
SNOMED-CT: Systematized Nomenclature of Medicine-Clinical Terms
UIMA: Unstructured Information Management Architecture
